# Whole-genome association analyses of sleep-disordered breathing phenotypes in the NHLBI TOPMed program

**DOI:** 10.1186/s13073-021-00917-8

**Published:** 2021-08-26

**Authors:** Brian E. Cade, Jiwon Lee, Tamar Sofer, Heming Wang, Man Zhang, Han Chen, Sina A. Gharib, Daniel J. Gottlieb, Xiuqing Guo, Jacqueline M. Lane, Jingjing Liang, Xihong Lin, Hao Mei, Sanjay R. Patel, Shaun M. Purcell, Richa Saxena, Neomi A. Shah, Daniel S. Evans, Craig L. Hanis, David R. Hillman, Sutapa Mukherjee, Lyle J. Palmer, Katie L. Stone, Gregory J. Tranah, NHLBI Trans-Omics for Precision Medicine (TOPMed) Consortium, Namiko Abe, Goncalo Abecasis, Christine Albert, Laura Almasy, Alvaro Alonso, Seth Ament, Peter Anderson, Pramod Anugu, Deborah Applebaum-Bowden, Dan Arking, Donna K. Arnett, Allison Ashley-Koch, Stella Aslibekyan, Tim Assimes, Paul Auer, Dimitrios Avramopoulos, John Barnard, Kathleen Barnes, R. Graham Barr, Emily Barron-Casella, Terri Beaty, Diane Becker, Lewis Becker, Rebecca Beer, Ferdouse Begum, Amber Beitelshees, Emelia Benjamin, Marcos Bezerra, Larry Bielak, Joshua Bis, Thomas Blackwell, John Blangero, Eric Boerwinkle, Ingrid Borecki, Donald W. Bowden, Russell Bowler, Jennifer Brody, Ulrich Broeckel, Jai Broome, Karen Bunting, Esteban Burchard, Brian Cade, Jonathan Cardwell, Cara Carty, Richard Casaburi, James Casella, Mark Chaffin, Christy Chang, Daniel Chasman, Sameer Chavan, Bo-Juen Chen, Wei-Min Chen, Yii-Der Ida Chen, Michael Cho, Seung Hoan Choi, Lee-Ming Chuang, Mina Chung, Elaine Cornell, Adolfo Correa, Carolyn Crandall, James Crapo, L. Adrienne Cupples, Joanne Curran, Jeffrey Curtis, Brian Custer, Coleen Damcott, Dawood Darbar, Sayantan Das, Sean David, Colleen Davis, Michelle Daya, Mariza de Andrade, Michael DeBaun, Ranjan Deka, Dawn DeMeo, Scott Devine, Ron Do, Qing Duan, Ravi Duggirala, Peter Durda, Susan Dutcher, Charles Eaton, Lynette Ekunwe, Patrick Ellinor, Leslie Emery, Charles Farber, Leanna Farnam, Tasha Fingerlin, Matthew Flickinger, Myriam Fornage, Nora Franceschini, Mao Fu, Stephanie M. Fullerton, Lucinda Fulton, Stacey Gabriel, Weiniu Gan, Yan Gao, Margery Gass, Bruce Gelb, Xiaoqi Priscilla Geng, Soren Germer, Chris Gignoux, Mark Gladwin, David Glahn, Stephanie Gogarten, Da-Wei Gong, Harald Goring, C. Charles Gu, Yue Guan, Xiuqing Guo, Jeff Haessler, Michael Hall, Daniel Harris, Nicola Hawley, Jiang He, Ben Heavner, Susan Heckbert, Ryan Hernandez, David Herrington, Craig Hersh, Bertha Hidalgo, James Hixson, John Hokanson, Elliott Hong, Karin Hoth, Chao Agnes Hsiung, Haley Huston, Chii Min Hwu, Marguerite Ryan Irvin, Rebecca Jackson, Deepti Jain, Cashell Jaquish, Min A. Jhun, Jill Johnsen, Andrew Johnson, Craig Johnson, Rich Johnston, Kimberly Jones, Hyun Min Kang, Robert Kaplan, Sharon Kardia, Sekar Kathiresan, Laura Kaufman, Shannon Kelly, Eimear Kenny, Michael Kessler, Alyna Khan, Greg Kinney, Barbara Konkle, Charles Kooperberg, Holly Kramer, Stephanie Krauter, Christoph Lange, Ethan Lange, Leslie Lange, Cathy Laurie, Cecelia Laurie, Meryl LeBoff, Jiwon Lee, Seunggeun Shawn Lee, Wen-Jane Lee, Jonathon LeFaive, David Levine, Dan Levy, Joshua Lewis, Yun Li, Honghuang Lin, Keng Han Lin, Xihong Lin, Simin Liu, Yongmei Liu, Ruth Loos, Steven Lubitz, Kathryn Lunetta, James Luo, Michael Mahaney, Barry Make, Ani Manichaikul, Jo Ann Manson, Lauren Margolin, Lisa Martin, Susan Mathai, Rasika Mathias, Patrick McArdle, Merry-Lynn McDonald, Sean McFarland, Stephen McGarvey, Hao Mei, Deborah A. Meyers, Julie Mikulla, Nancy Min, Mollie Minear, Ryan L. Minster, Braxton D. Mitchell, May E. Montasser, Solomon Musani, Stanford Mwasongwe, Josyf C. Mychaleckyj, Girish Nadkarni, Rakhi Naik, Take Naseri, Pradeep Natarajan, Sergei Nekhai, Deborah Nickerson, Kari North, Jeff O’Connell, Tim O’Connor, Heather Ochs-Balcom, Nicholette Palmer, James Pankow, George Papanicolaou, Margaret Parker, Afshin Parsa, Sara Penchev, Juan Manuel Peralta, Marco Perez, James Perry, Ulrike Peters, Patricia Peyser, Lawrence S. Phillips, Sam Phillips, Toni Pollin, Wendy Post, Julia Powers Becker, Meher Preethi Boorgula, Michael Preuss, Dmitry Prokopenko, Bruce Psaty, Pankaj Qasba, Dandi Qiao, Zhaohui Qin, Nicholas Rafaels, Laura Raffield, D. C. Rao, Laura Rasmussen-Torvik, Aakrosh Ratan, Susan Redline, Robert Reed, Elizabeth Regan, Alex Reiner, Muagututi‘a Sefuiva Reupena, Ken Rice, Stephen Rich, Dan Roden, Carolina Roselli, Jerome Rotter, Ingo Ruczinski, Pamela Russell, Sarah Ruuska, Kathleen Ryan, Phuwanat Sakornsakolpat, Shabnam Salimi, Steven Salzberg, Kevin Sandow, Vijay Sankaran, Christopher Scheller, Ellen Schmidt, Karen Schwander, David Schwartz, Frank Sciurba, Christine Seidman, Jonathan Seidman, Vivien Sheehan, Amol Shetty, Aniket Shetty, Wayne Hui-Heng Sheu, M. Benjamin Shoemaker, Brian Silver, Edwin Silverman, Jennifer Smith, Josh Smith, Nicholas Smith, Tanja Smith, Sylvia Smoller, Beverly Snively, Tamar Sofer, Nona Sotoodehnia, Adrienne Stilp, Elizabeth Streeten, Jessica Lasky Su, Yun Ju Sung, Jody Sylvia, Adam Szpiro, Carole Sztalryd, Daniel Taliun, Hua Tang, Margaret Taub, Kent D. Taylor, Simeon Taylor, Marilyn Telen, Timothy A. Thornton, Lesley Tinker, David Tirschwell, Hemant Tiwari, Russell Tracy, Michael Tsai, Dhananjay Vaidya, Peter VandeHaar, Ramachandran S. Vasan, Scott Vrieze, Tarik Walker, Robert Wallace, Avram Walts, Emily Wan, Fei Fei Wang, Heming Wang, Karol Watson, Daniel E. Weeks, Bruce Weir, Scott Weiss, Lu-Chen Weng, Cristen Willer, Kayleen Williams, L. Keoki Williams, Carla Wilson, James Wilson, Quenna Wong, Huichun Xu, Lisa Yanek, Ivana Yang, Rongze Yang, Norann Zaghloul, Maryam Zekavat, Yingze Zhang, Snow Xueyan Zhao, Wei Zhao, Xiuwen Zheng, Degui Zhi, Xiang Zhou, Xiaofeng Zhu, Michael Zody, Sebastian Zoellner, Gonçalo R. Abecasis, Eric A. Boerwinkle, Adolfo Correa, L. Adrienne Cupples, Robert C. Kaplan, Deborah A. Nickerson, Kari E. North, Bruce M. Psaty, Jerome I. Rotter, Stephen S. Rich, Russell P. Tracy, Ramachandran S. Vasan, James G. Wilson, Xiaofeng Zhu, Susan Redline, TOPMed Sleep Working Group, Brian Cade, Han Chen, Sina Gharib, Matthew Goodman, Daniel Gottlieb, Lauren Hale, Kristen Knutson, Diane Lauderdale, Jacqueline Lane, Jiwon Lee, Jingjing Liang, Xihong Lin, Yaowu Liu, Hao Mei, Braxton Mitchell, Debby Ngo, Jeff O’Connell, Heather Ochs-Balcom, Sanjay Patel, Shaun Purcell, Susan Redline, Jessica Rhodes, Richa Saxena, Neomi Shah, Tamar Sofer, Jae Hoon Sul, Shamil Sunyaev, Heming Wang, James Wilson, Man Zhang, Hufeng Zhou, Xiaofeng Zhu

**Affiliations:** 1 Division of Sleep and Circadian Disorders, Brigham and Women’s Hospital, Harvard Medical School, 221 Longwood Avenue, Boston, MA 02115 USA; 2 Division of Sleep Medicine, Harvard Medical School, Boston, MA 02115 USA; 3 Program in Medical and Population Genetics, Broad Institute, Cambridge, MA 02142 USA; 4 Department of Medicine, University of Maryland School of Medicine, Baltimore, MD 21201 USA; 5 Human Genetics Center, Department of Epidemiology, Human Genetics and Environmental Sciences, School of Public Health, The University of Texas Health Science Center at Houston, Houston, TX 77030 USA; 6 Center for Precision Health, School of Public Health and School of Biomedical Informatics, The University of Texas Health Science Center at Houston, Houston, TX 77030 USA; 7 Computational Medicine Core, Center for Lung Biology, UW Medicine Sleep Center, Division of Pulmonary, Critical Care and Sleep Medicine, University of Washington, Seattle, WA 98195 USA; 8 VA Boston Healthcare System, Boston, MA 02132 USA; 9 The Institute for Translational Genomics and Population Sciences, Department of Pediatrics, The Lundquist Institute for Biomedical Innovation at Harbor-UCLA Medical Center, Torrance, CA 90502 USA; 10 Center for Genomic Medicine and Department of Anesthesia, Pain, and Critical Care Medicine, Massachusetts General Hospital, Boston, MA 02114 USA; 11 Department of Population and Quantitative Health Sciences, School of Medicine, Case Western Reserve University, Cleveland, OH 44106 USA; 12 Department of Biostatistics, Harvard T.H. Chan School of Public Health, Boston, MA 02115 USA; 13 Department of Data Science, University of Mississippi Medical Center, Jackson, MS 29216 USA; 14 Division of Pulmonary, Allergy, and Critical Care Medicine, University of Pittsburgh, Pittsburgh, PA 15213 USA; 15 Division of Pulmonary, Critical Care and Sleep Medicine, Icahn School of Medicine at Mount Sinai, New York, NY 10029 USA; 16 California Pacific Medical Center Research Institute, San Francisco, CA 94107 USA; 17 Department of Pulmonary Physiology and Sleep Medicine, Sir Charles Gairdner Hospital, Perth, Western Australia 6009 Australia; 18 Sleep Health Service, Respiratory and Sleep Services, Southern Adelaide Local Health Network, Adelaide, South Australia Australia; 19 Adelaide Institute for Sleep Health, Flinders University, Adelaide, South Australia Australia; 20 School of Public Health, University of Adelaide, Adelaide, South Australia 5000 Australia; 21 Department of Biostatistics and Center for Statistical Genetics, University of Michigan School of Public Health, Ann Arbor, MI 48109 USA; 22 Human Genome Sequencing Center, Baylor College of Medicine, Houston, TX 77030 USA; 23 Department of Medicine, University of Mississippi Medical Center, Jackson, MS 39216 USA; 24 Jackson Heart Study, Jackson, MS 39216 USA; 25 Department of Biostatistics, Boston University School of Public Health, Boston, MA 02118 USA; 26 Framingham Heart Study, Framingham, MA 01702 USA; 27 Department of Epidemiology and Population Health, Albert Einstein College of Medicine, Bronx, New York, 10461 USA; 28 Department of Genome Sciences, University of Washington, Seattle, WA 98195 USA; 29 Northwest Genomics Center, Seattle, WA 98105 USA; 30 Department of Epidemiology and Carolina Center of Genome Sciences, University of North Carolina, Chapel Hill, NC 27514 USA; 31 Cardiovascular Health Study, Departments of Medicine, Epidemiology, and Health Services, University of Washington, Seattle, WA 98101 USA; 32 Kaiser Permanente Washington Health Research Institute, Seattle, WA 98101 USA; 33 Center for Public Health Genomics, University of Virginia, Charlottesville, VA 22908 USA; 34 Department of Pathology, University of Vermont, Colchester, VT 05405 USA; 35 Sections of Preventive Medicine and Epidemiology and Cardiology, Department of Medicine, Boston University School of Medicine, Boston, MA 02118 USA; 36 Department of Epidemiology, Boston University School of Public Health, Boston, MA 02118 USA; 37 Department of Physiology and Biophysics, University of Mississippi Medical Center, Jackson, MS 39216 USA; 38 Division of Pulmonary, Critical Care, and Sleep Medicine, Beth Israel Deaconess Medical Center, Boston, MA 02215 USA

**Keywords:** Sleep-disordered breathing, Sleep apnea, Whole-genome sequencing, WGS, Genome-wide association study, GWAS

## Abstract

**Background:**

Sleep-disordered breathing is a common disorder associated with significant morbidity. The genetic architecture of sleep-disordered breathing remains poorly understood. Through the NHLBI Trans-Omics for Precision Medicine (TOPMed) program, we performed the first whole-genome sequence analysis of sleep-disordered breathing.

**Methods:**

The study sample was comprised of 7988 individuals of diverse ancestry. Common-variant and pathway analyses included an additional 13,257 individuals. We examined five complementary traits describing different aspects of sleep-disordered breathing: the apnea-hypopnea index, average oxyhemoglobin desaturation per event, average and minimum oxyhemoglobin saturation across the sleep episode, and the percentage of sleep with oxyhemoglobin saturation < 90%. We adjusted for age, sex, BMI, study, and family structure using MMSKAT and EMMAX mixed linear model approaches. Additional bioinformatics analyses were performed with MetaXcan, GIGSEA, and ReMap.

**Results:**

We identified a multi-ethnic set-based rare-variant association (*p* = 3.48 × 10^−8^) on chromosome X with *ARMCX3*. Additional rare-variant associations include *ARMCX3-AS1*, *MRPS33*, and *C16orf90*. Novel common-variant loci were identified in the *NRG1* and *SLC45A2* regions, and previously associated loci in the *IL18RAP* and *ATP2B4* regions were associated with novel phenotypes. Transcription factor binding site enrichment identified associations with genes implicated with respiratory and craniofacial traits. Additional analyses identified significantly associated pathways.

**Conclusions:**

We have identified the first gene-based rare-variant associations with objectively measured sleep-disordered breathing traits. Our results increase the understanding of the genetic architecture of sleep-disordered breathing and highlight associations in genes that modulate lung development, inflammation, respiratory rhythmogenesis, and *HIF1A*-mediated hypoxic response.

**Supplementary Information:**

The online version contains supplementary material available at 10.1186/s13073-021-00917-8.

## Background

Sleep-disordered breathing (SDB) is a prevalent disorder associated with increased sleepiness, mortality, and morbidity from a wide range of cardiometabolic and other diseases [1, 2]. The most common type of SDB is obstructive sleep apnea (OSA), characterized by repeated airway collapse leading to intermittent hypoxemia and sleep disruption, that is increased in prevalence with older age and male sex [2]. An estimated 936 million adults aged 30–69 have mild to severe OSA worldwide [3]. The disease is heritable and appears to be multifactorial, reflecting variable contributions of abnormalities in ventilatory control, craniofacial anatomy, and adiposity [2, 4–7]. Sleep-related hypoxemia can also be due to central sleep apnea, a less common disorder, due to a lack of respiratory drive [8]. OSA is typically measured clinically using the apnea-hypopnea index, which counts the number of total (apnea) and partial (hypopnea) breathing cessations per hour of sleep. Due to an incomplete understanding of its molecular basis, the standard OSA treatment of continuous positive airway pressure (CPAP) only addresses the downstream manifestations of airway collapse through nightly use of pressurized air to the nasopharynx, a therapy that often is poorly tolerated. Therefore, there is a critical need to identify molecular pathways that could provide specific therapeutic targets. The need for overnight studies to phenotype SDB traits has limited the available sample size for genetic analyses, and only several common-frequency genome-wide analysis studies have been reported [9–11]. Increased statistical power may increase the genetic resolution of regions that may not be adequately tagged by current genotyping arrays due to population differences and/or reduced linkage disequilibrium with biologically relevant regions.

The Trans-Omics for Precision Medicine (TOPMed) program is an NIH National Heart, Lung, and Blood Institute program designed to improve the understanding of the biological processes that contribute to heart, lung, blood, and sleep disorders [12]. TOPMed has generated whole-genome sequencing (WGS) data on over 100,000 individuals from multiple cohorts at > 30× depth, including seven studies with objective assessment of SDB. A variant imputation server using TOPMed data also allows for high-quality imputation of non-sequenced genotype chip data [13]. A complementary initiative sponsored by the Centers for Common Disease Genomics (CCDG) of the NIH National Human Genome Research Institute has generated sequencing data from additional individuals in two TOPMed cohorts. These initiatives provide the ability to examine the genetics of SDB at unprecedented detail in African-Americans (AA), Asian-Americans (AsA), European-Americans/Australians (EA), and Hispanic/Latino-Americans (HA).

In this first genome-wide sequencing analysis of SDB, we examine the apnea-hypopnea index (AHI), the standard clinic metric of SDB, and four complementary measurements of overnight hypoxemia: average and minimum oxyhemoglobin saturation (SpO_2_) during sleep and the percent of the sleep recording with SpO_2_ < 90% (Per90), and the average desaturation per hypopnea event. These indices were chosen because of clinical relevance, high heritability, or prior significant GWAS findings [9, 11, 14]. We examined 7988 individuals with objectively measured SDB and WGS data in conjunction with data from 13,257 individuals with imputed genotype data.

## Methods

Each study had a protocol approved by its respective Institutional Review Board and participants provided informed consent. A study overview is provided in Additional file 2: Figure S1. There were two classes of data: “WGS studies” had WGS performed by the TOPMed program and, in some cases, in additional participants by the CCDG program (referred to as “WGS” studies); “Imputed studies” had array-based genotyping later imputed using the TOPMed imputation server (as described below). Some studies with WGS contributed imputed study data from additional array-based genotyped individuals. Ten studies were analyzed (Tables 1 and 2).

**Table 1:** Sample description for WGS cohorts

Population	Cohort	N	Age	Percent female	BMI	Apnea-hypopnea index 3%	AHI (percent < 5, 5–15, ≥ 15)	Average desaturation	Average SpO_**2**_	Minimum SpO_**2**_	Percent sleep under 90% SpO_**2**_
African-American	CFS*	505	38.65 (18.96)	56.4	32.44 (9.48)	6.85 (22.48)	43.4, 20.6, 36.0	3.62 (1.99)	94.49 (3.91)	84.76 (9.83)	4.79 (13.15)
	CHS	151	75.39 (4.35)	60.3	29.02 (5.08)	9.60 (16.96)	28.5, 36.4, 35.1	2.70 (1.74)	94.82 (2.19)	85.74 (5.35)	3.39 (9.63)
	JHS	575	63.47 (10.94)	64.9	31.8 (6.88)	10.69 (14.42)	24.7, 39.5, 35.8	3.54 (1.72)	94.77 (2.02)	84.30 (6.57)	2.97 (8.91)
	MESA	486	68.81 (9.07)	53.7	30.23 (5.68)	12.67 (20.56)	22.4, 32.9, 44.7	3.42 (2.10)	94.46 (1.99)	83.32 (7.98)	3.89 (9.49)
East Asian-American	MESA	229	67.89 (9.11)	49.8	24.28 (3.30)	14.96 (24.28)	21.8, 28.4, 49.8	3.72 (1.79)	94.92 (1.22)	83.23 (7.58)	2.25 (4.46)
European-American	ARIC	1028	62.28 (5.67)	53.1	28.72 (5.06)	8.64 (15.62)	34.6, 32.4, 33.0	2.35 (1.29)	94.57 (1.84)	85.95 (5.93)	2.92 (9.24)
	CFS*	485	43.23 (19.49)	50.5	30.81 (8.83)	7.09 (21.90)	44.7, 19.4, 35.9	3.29 (1.86)	93.67 (3.59)	85.55 (9.33)	4.66 (11.87)
	CHS	557	77.90 (4.34)	54.2	27.25 (4.44)	11.42 (15.54)	23.2, 38.1, 38.8	2.58 (1.34)	94.00 (2.00)	84.99 (5.67)	4.77 (12.28)
	FHS*	478	60.09 (8.54)	49.8	28.40 (5.06)	8.10 (14.28)	35.1, 35.1, 29.7	2.35 (1.27)	94.68 (2.04)	85.78 (6.25)	2.96 (9.18)
	MESA	698	68.53 (9.06)	53.2	27.91 (5.10)	12.18 (20.45)	21.6, 35.0, 43.4	3.11 (1.44)	93.96 (1.75)	83.49 (7.50)	4.27 (10.82)
Hispanic/Latino-American	HCHS/SOL	2339	46.27 (13.86)	60.5	30.23 (6.44)	2.03 (6.30)	68.9, 19.5, 11.6	N/A	96.42 (0.99)	87.04 (5.92)	0.88 (3.63)
	MESA	456	68.49 (9.27)	53.3	30.08 (5.46)	16.31 (22.53)	17.1, 28.3, 54.6	3.62 (2.12)	94.33 (1.60)	81.59 (9.32)	3.80 (7.64)

Seven studies contributed 7988 individuals with WGS in TOPMed Freeze 6a and objectively measured phenotypes (1717 African-Americans, 229 Asian-Americans, 3246 European-Americans, 2796 Hispanic/Latino-Americans). The overall sample had a mean age of 57.7 and was 56.1% female. Values are displayed as mean (SD), except for the skewed apnea-hypopnea index, which is displayed as median (IQR). Sample size N reflects individuals with non-missing AHI and covariate values. *Family cohort

**Table 2:** Sample description for imputed genotype chip cohorts

Population	Cohort	N	Age	Percent female	BMI	Apnea-hypopnea index 3%	AHI (percent < 5, 5–15, ≥ 15)	Average desaturation	Average SpO_**2**_	Minimum SpO_**2**_	Percent sleep under 90% SpO_**2**_
African-American	CFS*	225	35.46 (20.32)	56.4	29.97 (10.09)	3.99 (10.55)	55.1, 23.1, 21.8	2.90 (1.09)	94.65 (4.01)	88.17 (9.60)	5.20 (16.01)
European-American, Australian	ARIC	631	62.74 (5.72)	49.4	29.15 (5.23)	9.15 (15.02)	29.3, 37.9, 32.8	2.50 (1.73)	94.32 (2.15)	85.17 (6.17)	4.12 (11.76)
	CFS*	218	37.57 (18.66)	56.9	28.76 (8.11)	3.4 (10.59)	57.8, 22.5, 19.7	2.30 (1.11)	94.09 (3.35)	88.81 (7.80)	3.26 (12.79)
	CHS	365	77.44 (4.65)	64.9	27.10 (4.41)	10.50 (15.14)	25.8, 39.2, 35.1	2.63 (1.57)	94.41 (1.91)	84.87 (5.96)	3.93 (11.89)
	FHS*	192	57.45 (9.68)	51.0	28.87 (5.16)	7.30 (14.38)	38.0, 31.8, 30.2	2.42 (1.51)	94.73 (1.80)	85.76 (5.46)	2.82 (8.38)
	MrOS	2181	76.65 (5.60)	0.0	27.21 (3.75)	13.00 (18.00)	18.9, 36.1, 45.0	3.54 (1.48)	93.85 (1.73)	84.39 (5.88)	4.40 (9.95)
	WASHS	1508	52.29 (13.71)	40.9	31.84 (7.93)	7.24 (15.37)	40.1, 31.1, 28.8	3.56 (2.00)	94.56 (2.38)	84.61 (7.86)	5.44 (13.82)
Hispanic, Latino-American	HCHS, SOL	7155	46.10 (13.81)	57.8	29.68 (5.86)	2.00 (6.15)	69.1, 19.3, 11.6	N, A	96.46 (0.95)	87.06 (6.11)	0.83 (2.99)
	Starr	782	52.34 (11.29)	71.9	32.15 (6.78)	10.35 (17.18)	31.5, 31.5, 37.1	N, A	94.65 (2.09)	85.78 (7.50)	2.83 (8.79)

Eight studies contributed 13,257 individuals with genomic data imputed with a TOPMed Freeze 5b reference panel and objectively measured phenotypes (225 African-Americans, 5095 European-Americans, 7937 Hispanic/Latino-Americans). ARIC, CFS, CHS, FHS, and HCHS/SOL imputed genomic data reflect individuals without available sequencing in TOPMed Freeze 6. The overall sample had a mean age of 53.7 and was 46.9% female. Values are displayed as mean (SD), except for the skewed apnea-hypopnea Index, which is displayed as median (IQR). Sample size N reflects individuals with non-missing AHI and covariate values. *Family cohort

### WGS studies

The Atherosclerosis Risk in Communities Study (ARIC), the Cardiovascular Health Study (CHS), and the Framingham Heart Study Offspring Cohort (FHS) included individuals who participated in the Sleep Heart Health Study (SHHS), who underwent polysomnography (PSG) between 1995 and 1998 using the Compumedics PS-2 system [15–18]. These samples included 1028 EAs from ARIC, 151 AAs and 557 EAs from CHS, and 478 EAs from FHS.

The Multi-Ethnic Study of Atherosclerosis (MESA) is investigating the risk factors for clinical cardiovascular disease [19]. PSG was obtained between 2010 and 2013 using the Compumedics Somte system [20]. This analysis includes data from 698 EAs, 486 AAs, 456 HAs, and 229 AsAs.

The Cleveland Family Study (CFS) was designed to investigate the familial basis of SDB, with four visits occurring from 1990 to 2006 [21]. Sleep was assessed either in a clinical research center using full PSG (Compumedics E series) (visit 4) or in the latest available prior examination using an in-home sleep apnea testing device (Edentrace). Data were analyzed from 505 AAs and 485 EAs (339 AAs and 234 EAs with full PSG data).

The Hispanic Community Health Study/Study of Latinos (HCHS/SOL) is studying multiple health conditions in HAs [22, 23]. Home sleep apnea testing was performed during the baseline examination (2008–2011) using the ARES Unicorder 5.2, a validated device including a forehead-based reflectance oximeter, a nasal pressure cannula and pressure transducer, an accelerometer, and a microphone [24]. Two thousand three hundred thirty-nine individuals provided data.

The Jackson Heart Study (JHS) is investigating cardiovascular disease in AAs [25]. An in-home sleep study was performed from 2012 to 2016 using a validated type 3 sleep apnea testing device (Embla Embletta Gold) [26, 27]. Five hundred seventy-five individuals contributed data.

### Imputed genotype studies

The Osteoporotic Fractures in Men Study (MrOS) is a multi-center cohort study initially designed to examine the risk factors for osteoporosis, fractures, and prostate cancer in older males [28, 29]. An ancillary study (MrOS Sleep; 2003–2005) focused on outcomes of sleep disturbances used PSG and nearly identical procedures as in MESA (Compumedics Safiro system) [30]. Two thousand one hundred eighty-one EA individuals were included, with genotyping performed using the Illumina Human Omni 1 Quad v1-0 H array.

The Starr County Health Studies (Starr) investigates the risk factors for diabetes in Mexican-Americans [31, 32]. An in-home sleep apnea study occurred between 2010 and 2014 using a validated instrument that records finger pulse oximetry, actigraphy, body position, and peripheral arterial tonometry (Itamar-Medical WatchPAT-200) [33]. Seven hundred eighty-two HA individuals were studied, using Affymetrix 6.0 genotyping data.

The Western Australian Sleep Health Study (WASHS) is a clinic-based study focused on the epidemiology and genetics of SDB [34]. PSG was obtained from 1508 European-ancestry patients (91% referred for SDB evaluation) from 2006 to 2010 (Compumedics Series E). Genotyping was performed using the Illumina Omni 2.5 array.

Imputed genotype data were available for additional members of the TOPMed cohorts described above. Study/population combinations with fewer than 100 individuals were excluded. ARIC contributed an additional 631 EA individuals (Affymetrix 6.0; dbGaP phg000035.v1.p1). CFS contributed 225 AA and 218 EA individuals (Affymetrix 6.0; Illumina OmniExpress+Exome, Exome, and IBC). CHS contributed 365 individuals (Illumina CNV370 and IBC; phg000135.v1.p1 and phg000077.v1.p1). FHS contributed 192 EA individuals (Affymetrix 500 k; phg000006.v7). HCHS/SOL contributed 7155 HA individuals (Illumina Omni 2.5; phg000663.v1).

### Phenotype and covariate definitions

We examined several SDB measures, including specific measures of OSA: AHI (number of apneas plus hypopneas per hour of sleep, with a minimum 3% desaturation per event) and average oxyhemoglobin desaturation per apnea or hypopnea, and measures of SDB severity [14]: average and minimum SpO_2_ and the percentage of the night with SpO_2_ < 90% (Per90). Apart from WASHS, all sleep data were scored by blinded scorers at one central Sleep Reading Center with high levels of scorer reliability using well-defined procedures [35]. The AHI reflected all events. We did not attempt to disentangle the apnea-hypopnea index from central versus obstructive sleep apnea events, due to the relatively low prevalence of central sleep apnea (< 2%) in these largely community-based studies [36, 37] (some of which are enriched with snorers) and the complexities of classifying mixed events. We adjusted for age, age^2^, sex, age × sex, body mass index (BMI), and BMI^2^ due to known age and sex effects, some of which are non-linearly associated with outcomes, and our goal of identifying obesity-independent loci. Age and BMI were obtained at the time of the sleep recording. We adjusted for BMI as over half of the AHI trait heritability is attributable to factors other than obesity as measured by the BMI and our goal was to identify associations with other mechanistic pathways (e.g., ventilatory control) that could indicate novel future targets. Phenotype analyses were pooled within populations to aggregate very rare variants for testing and therefore further adjusted for study. Population assignments were based on self-report, in accordance with other research from TOPMed and other consortia. AsA and EA-identifying individuals with population principal components > 5 standard deviations [38] from applicable 1000 Genomes and Human Genome Diversity Project super-populations were excluded. We used a two-stage procedure to rank-normalize the phenotypes adjusted for covariates [39]. Cryptic relatedness and population substructure were controlled for using linear mixed models. Genomic control was applied to population-specific results (or cohort-specific imputed genotype results).

### WGS and genotyping

Sequence data were derived from the TOPMed Freeze 6a release, jointly called by the TOPMed Informatics Research Center at the University of Michigan (http://github.com/statgen/topmed_variant_calling). The methodology was described elsewhere [12]. In brief, WGS was performed at the Broad Institute (ARIC, FHS, MESA), Baylor College of Medicine (ARIC, CHS, HCHS/SOL), and the University of Washington (CFS, JHS). Additional ARIC and HCHS/SOL WGS funded by CCDG (https://www.genome.gov/27563570) and performed at Baylor College of Medicine were included in the jointly called data. TOPMed and CCDG calling pipelines have functionally equivalent outcomes despite data processing differences (as detailed in [40]). WGS data were merged and normalized; inferred sequence contamination was identified; and SNPs and small indels were detected (structural variants are not currently available). Lower quality variants were excluded using Mendelian consistency checks. Variants were aligned to Build 38 and annotated using snpEff 4.3 t [41]. We excluded variants with < 10× depth or > 5% missingness, leaving 152.7 million polymorphic variants in 7988 individuals with SDB phenotypes. Up to 22,030,888 variants from individuals with sequencing were tested in the GWAS analyses, following filtering for quality control and minor allele frequencies.

Genotype data were imputed using the TOPMed Imputation Server [13] using a Freeze 5b (Build 38) template. Forward strand checks were performed using the Strand database and the Haplotype Reference Consortium imputation preparation script (https://www.well.ox.ac.uk/~wrayner/tools/) and confirmed using Ensembl variant allele checks and internal QC performed on the server. Study-level data were imputed separately. Analyses on variants with r^2^ score > 0.5 were therefore performed separately for each study. Up to 22,105,437 variants from individuals with imputed data were tested in the GWAS analyses, following filtering for quality control, imputation r^2^, and minor allele frequencies.

### Statistical analyses

Single and grouped variant analyses were performed using EMMAX and MMSKAT, both within the EPACTS suite (v3.3) [42]. WGS genetic relatedness matrices (GRM) were constructed using autosomal variants (MAF > 0.1%) following a comparison of EPACTS point-wise heritability estimates of the AHI using different minimal MAFs. A grid search identified optimal GRM parameters with imputed data (MAF > 0.5%, r^2^ > 0.90) using 929 ARIC individuals with imputation and WGS data. Log_10_ *P*-values using identical association test parameters had a Spearman’s ρ correlation of 0.951 between WGS and imputed data. Matrices were constructed separately for each study + population combination (due to potentially differential imputation coverage).

Gene-based group sets considered Ensembl-defined non-pseudogenes expressed in any GTEx v7 tissue. Variants needed to clear a series of frequency, regional, functional class, and presumed functionality score filters in order to test a gene using its most biologically plausible variants. Variants could have a maximum minor allele frequency of 5%. Regions were largely exon-based. We also included variants located within experimentally derived promoter regions and Ensembl-derived Tarbase miRNA binding sites; and regulatory variants located within 1000 bases of a particular gene, including ChIP-seq determined transcription factor binding sites (TFBS), and Ensembl-derived CTCF, TFBS, and promoter sites [43–45]. Variants from a subset of 19 snpEff gene-based annotation functional classes (e.g., missense or nonsense, but not synonymous mutations) were considered. Finally, group set variants passing these prior filters were additionally filtered for the plausibility of biological function by requiring either a FATHMM-XF score > 0.5 or a CDTS < 1% constrained region score [46, 47]. Exonic variants could alternatively have a PrimateAI score > 0.803 or a Havrilla et al. < 1% constrained coding region score [48, 49].

Gene-based tests considered variants in WGS-only data. Pooled (across cohort) analyses were performed within each population in order to aggregate information on very rare variants across studies. Combined population results were obtained through meta-analysis of *p*-values weighted by sample size (due to potentially different MAF spectra driven by population demography). A significance level of *p* < 4.51 × 10^−8^ was used, reflecting a Bonferroni adjustment for all genes tested across all phenotype and population configurations.

A second set-based analysis was designed to query for TFBS annotation enrichment [50]. We performed 250-base pair sliding window analyses (to improve power by aggregating additional variants beyond an approximate ChIP-seq peak width of 100 base pairs). We filtered for variants with either a FATHMM-XF score > 0.5 or a CDTS 1% score with no MAF cut-offs and meta-analyzed MMSKAT results across the 4 populations, noting windows with *p*-values < 0.01. These intervals were tested for enrichment of ChIP-seq coordinates with at least 50% physical overlap for up to 437 transcription factors using ReMap 2018 v1.2 [51].

Single-variant EMMAX tests examined common variants (MAF > 0.5%). Meta-analysis across populations (and imputed genotype studies) used METAL with genomic control [52]. We performed bidirectional discovery and replication using the WGS and imputed samples (noting the high genomic resolution in the WGS samples and the higher sample size in the imputed data). We report results including at least 1000 individuals in discovery analyses, discovery association *p*-values < 1 × 10^−5^ and replication association *p*-values < 0.05. Therefore, no population-specific discovery analyses of Asian-Americans were performed. Multi-ethnic analyses included a minimum of two populations where a variant cleared minimum MAF and imputation quality (for chip-based results) criteria. Significance was defined as *p* < 1 × 10^−8^ in joint analyses, reflecting adjustment for five correlated phenotypes (Additional file 1: Table S3). We performed MetaXcan imputed GTEx gene expression analyses using joint EA results in selected tissues relevant to SDB and GIGSEA pathway analyses of MetaXcan output in whole blood (to maximize power), with empirical *p*-values incorporating 10,000 permutations [53, 54]. Bioinformatics annotations of single-variant results (Additional file 1: Table S7) include significant eQTL associations from GTEx v7, and overlapping promoter and enhancer coordinates derived from Roadmap Epigenomics, BLUEPRINT, and Vermunt et al. brain tissues (enhancers only) [55–58]. Lookups of potentially druggable genes as defined within DGIdb, a database of 56,000 drug-gene interactions from over 30 literature sources, were performed using the GeneCards suite [59, 60].

## Results

### Study sample

A study overview is provided in Additional file 2: Figure S1. Tables 1 and 2 provide a summary of the study samples and SDB traits analyzed using WGS and imputed genotypes, respectively. In total, there were 21,244 individuals (1942 AAs, 229 AsAs, 8341 EAs, and 10732 HAs). Median AHI levels ranged from mildly to moderately elevated, reflecting the age range and sex distribution of each cohort. Pairwise correlations of phenotypes and covariates are provided in Additional file 1: Table S3.

### Gene-based results

Gene-based rare-variant results are presented in Table 3 (for meta-analyzed results across multiple populations) and in Table 4 (for secondary population-specific results). Collectively, we identified four significantly associated genes (Bonferroni *p* < 4.51 × 10^−8^). *ARMCX3*, identified in the multiple-population analysis, is an X-linked protein-coding that was associated with average desaturation (*p* = 5.29 × 10^−8^). Two protein-coding genes were identified in population-specific analyses of Per90: *MRPS33* (*p* = 1.22 × 10^−9^) and *C16orf90* (*p* = 1.36 × 10^−8^). We identified 12 suggestively associated genes (p ≤ 4.22 × 10^−7^). Three genes are druggable [59, 60]. Nominally significant results (*p* < 0.01) and additional details are presented in Additional file 1: Tables S4 and S5. A list of individual variants comprising each gene is provided in Additional file 1: Table S6.

**Table 3:** Lead gene-based multiple-population results

Phenotype	Sex	Gene	B38 positions	P	N	Variants	Population P	Population N	Population variants
Avg desaturation	All	*ARMCX3*	X:101,623,082–101,625,765	**3.48 × 10**^**−8**^	5222	41	0.220, 0.179, 2.17 × 10^−6^, 8.93 × 10^−4^	1545; 227; 2994; 456	8, 5, 24, 9
	All	*ARMCX3-AS1*	X:101,623,082–101,625,153	**3.49 × 10**^**−8**^	5222	38	0.225, 0.179, 2.19 × 10^−6^, 8.20 × 10^−4^	1545; 227; 2994; 456	7, 5, 23, 8
Per90	All	*OR5K2*	3:98,497,633–98,498,634	2.55 × 10^−7^	7986	7	0.143, 0.440, 4.14 × 10^−2^, 2.74 × 10^−7^	1712; 229; 3,242; 2803	4, 2, 1, 1
Per90	Females	*ZZEF1*	17:4,004,409–4,144,018	4.22 × 10^−7^	4485	236	0.634, 0.337, 5.03 × 10^−4^, 3.05 × 10^−5^	1009; 114; 1702; 1660	85, 16, 87, 131

Lead MMSKAT gene-based results meta-analyzed across populations within one order of magnitude of significance (*p* < 4.51 × 10^−8^) are shown. Population-specific information for each gene is displayed in the latter columns for AA, AsA, EA, and HA, respectively. Individual populations varied in the number of polymorphic variants available for testing (e.g., due to singletons or excessively common variants). *ARMCX3-AS1* is a RNA gene that is anti-sense to the protein-coding *ARMCX3* gene*.* Full results for genes with *p* < 0.01, including Ensembl-derived gene biotypes and descriptions, are provided in Additional file 1: Table S4. A list of individual variants comprising each gene is provided in Additional file 1: Table S6

**Table 4:** Lead gene-based population-specific results

Phenotype	Model	Gene	B38 positions	N	Variants	Singletons	P
Per90	HA	*LINC01277*	6:142,985,371–143,010,415	2803	2	0	5.02 × 10^−8^
		*OR5K2*	3:98,497,633–98,498,634	2803	1	0	2.74 × 10^−7^
	AA females	S100A16*	1:153,607,528–153,616,353	1009	1	1	2.07 × 10^−7^
		*CSMD2-AS1*	1:33,867,977–33,885,456	1009	1	1	2.07 × 10^−7^
	EA females	*MRPS33*	7:141,006,422–141,014,911	1702	9	8	**1.22 × 10**^−**9**^
		*LINC01811*	3:34,170,921–34,558,474	1702	6	5	9.71 × 10^−8^
		NELFCD*	20:58,980,722–58,995,761	1702	12	10	3.32 × 10^−7^
		SLC22A8*	11:62,988,399–63,015,986	1702	3	3	3.58 × 10^−7^
	HA females	*AL132709.1*	14:101,077,452–101,077,578	1660	2	0	1.41 × 10^−7^
		*EPHX4*	1:92,029,443–92,063,474	1660	12	10	3.48 × 10^−7^
	HA males	*C16orf90*	16:3,493,483–3,496,479	1143	6	3	**1.36 × 10**^−**8**^
		*TVP23B*	17:18,781,270–18,806,714	1143	4	4	2.53 × 10^−7^
		*IPCEF1*	6:154,154,536–154,356,890	1143	10	8	4.07 × 10^−7^

Lead MMSKAT gene-based population-specific associations within one order of magnitude of significance (*p* < 4.51 × 10^−8^) are shown. The Variants column indicates the number of filtered polymorphic variants with minor allele frequency < 5% available for testing, a portion of which were singletons. *Druggable gene [59, 60]. Full results for genes with *p* < 0.01, including descriptions, are provided in Additional file 1: Table S5. A list of individual variants comprising each gene is provided in Additional file 1: Table S6

### Single-variant results

**Fig. 1:**
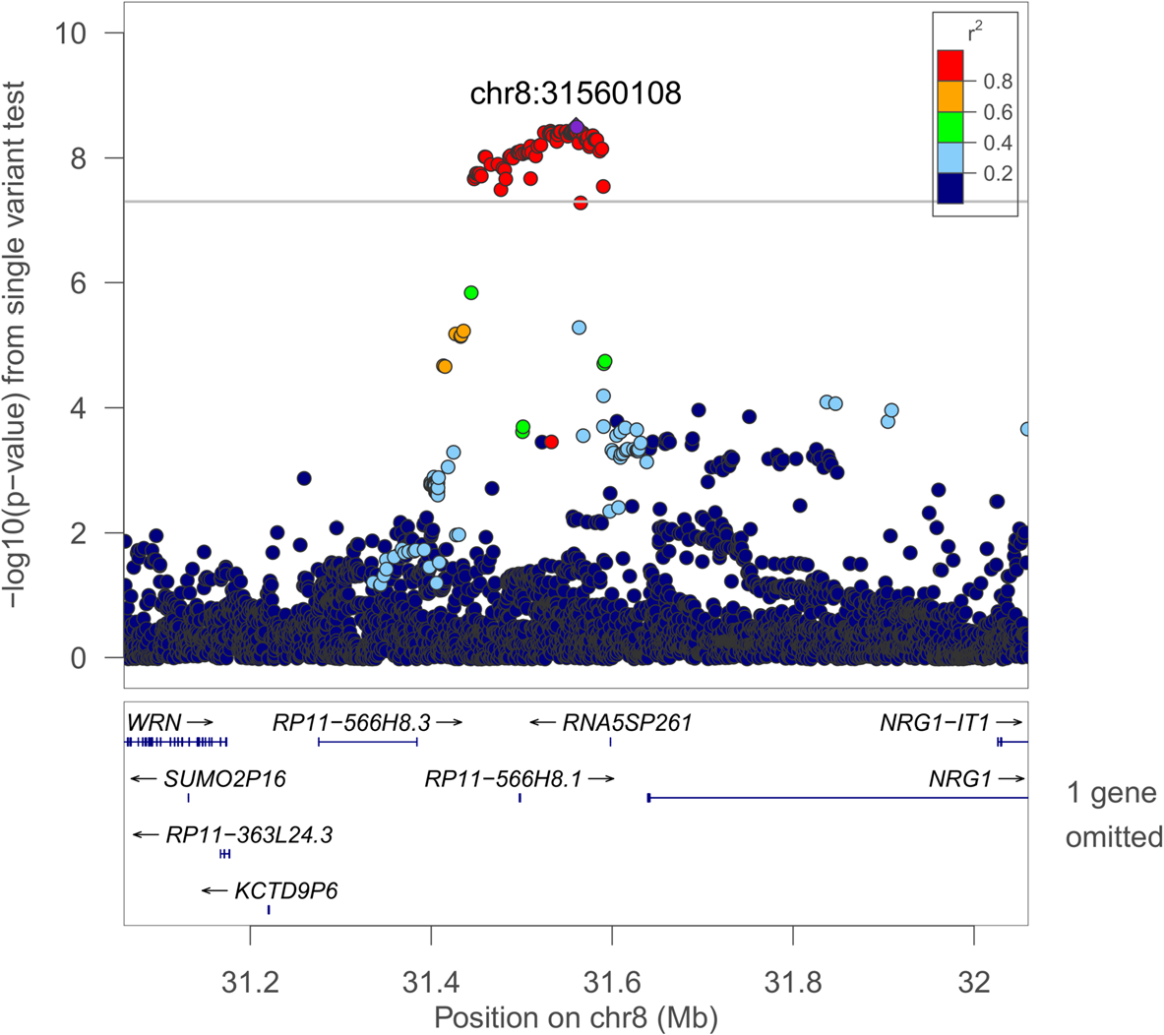
Regional plot of the rs35447033 association with AHI in European-ancestry individuals. Joint WGS and imputed results are shown, using Build 38 coordinates on the X-axis. Log-transformed *p*-values are shown on the Y-axis. Variant colors indicate the degree of linkage disequilibrium with the lead variant rs35447033

**Fig. 2:**
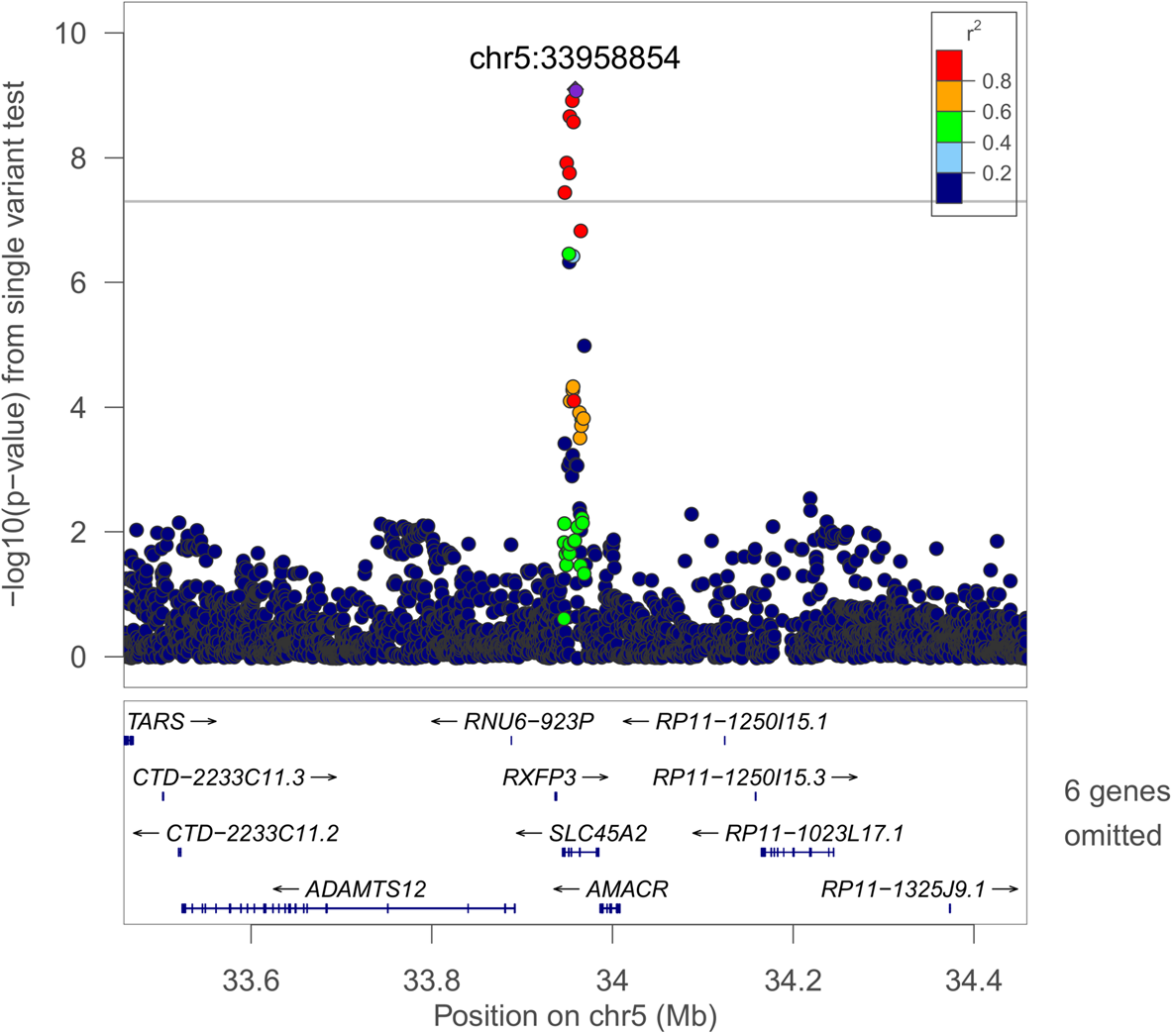
Regional plot of the rs28777 association with average SpO_2_ in European-ancestry individuals. Joint WGS and imputed results are shown, using Build 38 coordinates on the X-axis. Log-transformed *p*-values are shown on the Y-axis. Variant colors indicate the degree of linkage disequilibrium with the lead variant rs28777

We identified four genome-level significant loci in single-variant analyses (MAF > 0.5%; *p* < 1.0 × 10^−8^; Table 5). In multiple-population analyses, the 2q12 locus (rs77375846; *IL18RAP*) was associated with average event desaturation in a multiple-population analysis (combined *p* = 1.57 × 10^−9^) and minimum SpO_2_ (consistent with a previous report [10]). Two novel population-specific loci were identified. The 8p12 locus (rs35447033, *NRG1*) was associated with AHI in EAs (combined *p* = 3.02 × 10^−9^, Fig. 1). The 5p13 locus (rs28777; *SLC45A2*) was associated with average SpO_2_ in EAs (combined *p* = 8.08 × 10^−10^, Fig. 2). In HAs, the 1q32 locus (rs116133558; *ATP2B4*) was associated with Per90 (combined *p* = 3.51 × 10^−10^) and with average SpO_2_ (as previously identified [9]). Twelve additional regions were suggestively associated (*p* < 1.0 × 10^−7^). Additional file 1: Table S7 provides additional context for all variants in these loci (*p* < 1.0 × 10^−7^), including imputation quality, significant eQTLs, and overlap with epigenetic regions. Lookups of loci that we have identified in prior publications [9–11] are provided in Additional file 1: Table S8. Manhattan and QQ plots corresponding to the significant associations are provided in Additional file 2: Figures S2–S5. GWAS summary statistics have been posted to the Broad Institute Sleep Disorders Research Portal (https://sleep.hugeamp.org/).

**Table 5:** Lead single-variant analysis results

Region	Phenotype	Model	SNP	WGS/Chip N	CAF	WGS beta (SE)	WGS P	Chip beta (SE)	Chip P	Combined beta (SE)	Combined P
*2q12.1: IL18RAP*	Avg desaturation	All	rs77375846 C	4995, 4838	0.028–0.129	−0.152 (0.049)	1.87 × 10^−3^	−0.264 (0.049)	5.97 × 10^−8^	−0.208 (0.035)	**1.57 × 10**^**−9**^
*2q33.3: PPIAP68*	Avg desaturation	All	rs60132122 T	5222, 4838	0.308–0.637	0.062 (0.031)	0.043	0.195 (0.034)	6.26 × 10^−9^	0.122 (0.023)	6.49 × 10^−8^
*11q12.2: MS4A15*	Avg SpO_2_	All	rs4939452 C	7929, 13197	0.347–0.524	0.066 (0.023)	4.34 × 10^−3^	0.063 (0.014)	3.29 × 10^−6^	0.064 (0.012)	4.87 × 10^−8^
*18q12.3: LINC00907*	Avg SpO_2_	All	rs187860354 G	4500, 7391	0.006–0.022	0.442 (0.146)	2.36 × 10^−3^	0.432 (0.097)	8.53 × 10^−6^	0.436 (0.081)	7.04 × 10^−8^
*2q12.1: IL18RAP*	Min SpO_2_	All	rs138895820 G	7705, 13194	0.025–0.131	0.510 (0.184)	5.58 × 10^−3^	0.654 (0.128)	3.36 × 10^−7^	0.607 (0.105)	**7.93 × 10**^**−9**^
*10p12.31: NEBL*	Min SpO_2_	Females	rs11453507 CA	4450, 6202	0.138–0.514	0.651 (0.140)	3.34 × 10^−6^	0.338 (0.102)	8.63 × 10^−4^	0.446 (0.082)	5.73 × 10^−8^
*12q21.2: LINC024064*	Min SpO_2_	Females	rs2176909 T	4450, 6202	0.724–0.930	0.828 (0.157)	1.38 × 10^−7^	0.319 (0.116)	5.77 × 10^−3^	0.498 (0.093)	9.06 × 10^−8^
*5p13.3: C5orf22*	AHI	Males	rs10940956 A	3502, 7043	0.470–0.759	0.930 (0.422)	2.74 × 10^−2^	1.430 (0.269)	1.09 × 10^−7^	1.285 (0.227)	1.48 × 10^−8^
*9p22.1: DENND4C*	AHI	AA	rs111654000 A	1717, 225	0.016–0.018	−11.240 (2.268)	7.18 × 10^−7^	−18.110 (6.724)	7.07 × 10^−3^	−11.942 (2.149)	2.74 × 10^−8^
*1q31.2: AL954650.1*	AHI	AA	chr1:191965014_G/A A	1717, 225	0.286–0.301	3.078 (0.641)	1.56 × 10^−6^	5.080 (1.759)	3.88 × 10^−3^	3.313 (0.602)	3.75 × 10^−8^
*8p12: AC068672.1, NRG1*	AHI	EA	rs35447033 T	3246, 5095	0.060–0.094	2.247 (0.621)	2.95 × 10^−4^	2.453 (0.521)	2.54 × 10^−6^	2.368 (0.399)	**3.02 × 10**^**−9**^
*5p13.2: SLC45A2*	Avg SpO_2_	EA	rs28777 A	3201, 5024	0.885–0.969	−0.526 (0.133)	8.00 × 10^−5^	−0.454 (0.096)	2.23 × 10^−6^	−0.478 (0.078)	**8.08 × 10**^**−10**^
*1q32.1: ATP2B4*	Avg SpO_2_	HA	rs116133558 T	2803, 7956	0.006–0.014	0.371 (0.120)	2.08 × 10^−3^	0.294 (0.062)	2.15 × 10^−6^	0.310 (0.055)	1.88 × 10^−8^
*1q23.3: intergenic (RNU6-755P)*	Min SpO_2_	HA	rs140743827 A	2803, 7174	0.017–0.020	−1.502 (0.593)	1.13 × 10^−2^	−1.770 (0.367)	1.42 × 10^−6^	−1.696 (0.312)	5.51 × 10^−8^
*1q32.1: ATP2B4*	Per90	HA	rs116133558 T	2803, 7956	0.006–0.014	−1.005 (0.450)	2.54 × 10^−2^	−1.218 (0.207)	4.15 × 10^−9^	−1.181 (0.188)	**3.51 × 10**^**−10**^
*11p11.2: intergenic (AC104010.1)*	Avg SpO_2_	HA males	chr11:44652095_TC/T T	1143, 3024	0.007–0.008	0.686 (0.248)	5.65 × 10^−3^	0.710 (0.154)	3.83 × 10^−6^	0.703 (0.131)	7.25 × 10^−8^
*10q22.1:HK1*	Min SpO_2_	EA males	rs17476364 C	1523, 3650	0.072–0.115	1.215 (0.392)	1.94 × 10^−3^	1.099 (0.235)	2.81 × 10^−6^	1.129 (0.201)	2.01 × 10^−8^
*8q23.2: KCNV1*	Min SpO_2_	EA males	rs58365105 A	1523, 3650	0.007–0.026	−2.878 (0.864)	8.65 × 10^−4^	−2.406 (0.540)	8.36 × 10^−6^	−2.539 (0.458)	2.96 × 10^−8^
*2q35: AC019211.1*	Per90	EA males	chr2:220369683_G/A A	1540, 187	0.005–0.006	12.280 (2.431)	4.38 × 10^−7^	17.505 (7.989)	2.85 × 10^−2^	12.723 (2.326)	4.48 × 10^−8^

Lead EMMAX single-variant associations within one order of magnitude of significance (combined *p* < 1.00 × 10^−8^) and with replication evidence (*p* < 0.05) are shown. Full results for all variants in each locus with *p* < 1.00 × 10^−7^, including additional associations with secondary models, and metadata and annotations, are provided in Additional file 1: Table S7

### MetaXcan imputed gene expression and GIGSEA pathway analyses

We used joint WGS and imputed EA results to impute associations with gene expression levels using a MetaXcan framework for six tissues (subcutaneous and visceral omentum adipose, lung, monocytes, skeletal muscle, and whole blood). No individual tests reached Bonferroni significance (*p* < 2.60 × 10^−7^; Additional file 1: Table S9). Genes that were observed in the top 10 results across the varied analyses (Additional file 1: Table S10) included *ZNF83* (15 instances) and *CHRNE* (13 instances).

Whole blood MetaXcan results (with the largest sample size) were further evaluated in GIGSEA-based pathway analyses. KEGG pathway results are shown in Additional file 1: Table S11. The most significantly associated pathway was KEGG_STEROID_HORMONE_BIOSYNTHESIS (average SpO_2_ empirical *p*-value = 7.00 × 10^−4^). KEGG_RIG_I_LIKE_RECEPTOR_SIGNALING_PATHWAY was observed in the top 10 results for four of the five phenotypes. Gene-centric transcription factor binding site (TFBS) enrichment analysis results are presented in Additional file 1: Table S12. V$PEA3_Q6 (*ETV4*) was the most significantly associated TFBS (average desaturation empirical *p*-value = 3.00 × 10^−4^) and was the strongest association for AHI and minimum SpO_2_ (empirical *p*-values 0.002 and 0.001, respectively). The most significant miRNA binding site enrichment analysis association was GCATTTG,MIR-105 (average SpO_2_ *p* = 0.002; Additional file 1: Table S13). AGGCACT,MIR-515-3P (the strongest AHI association, *p* = 0.009) was observed in the top ten results for four phenotypes.

### ChIP-seq transcription factor binding site interval enrichment

We performed a sliding window analysis to examine enriched intervals containing ChIP-seq derived coordinates for up to 437 transcription factors (Table 6, Additional file 1: Table S14). *FOXP2* TFBS were consistently the most enriched for all phenotypes. Other notable transcription factors in the top 5 included *EGR1*, *KDM4B*, *KDM6B*, and *TP63*. *KDM4B* and *KDM6B* are druggable [59, 60]. Leading sliding window results are provided in Additional file 1: Table S15.

**Table 6:** Transcription factor binding site interval enrichment results

Phenotype	Transcription factor	# Observed overlap	# Expected overlap	−log10 (E-value)
AHI	*FOXP2*	588	36.20	473.99
	*KDM6B*	630	51.58	435.29
	*THAP1*	505	31.89	402.07
	*KLF9*	745	91.81	395.52
	*TP63*	997	182.22	383.85
Average desaturation	*FOXP2*	493	22.32	460.00
	*THAP1*	439	19.55	412.76
	*UBTF*	489	28.20	407.50
	*TP63*	788	109.36	382.89
	*KDM6B*	482	30.98	380.39
Average SpO_2_	*FOXP2*	582	35.87	468.89
	*KDM6B*	613	51.21	418.65
	*EGR1*	664	66.76	404.83
	*UBTF*	574	46.35	399.91
	*KDM4B*	489	29.56	398.10
Min SpO_2_	*FOXP2*	561	35.57	445.57
	*THAP1*	515	31.32	417.89
	*KDM6B*	569	50.87	373.41
	*UBTF*	536	45.99	360.56
	*EGR1*	602	66.25	346.03
Per90	*FOXP2*	689	39.05	578.42
	*KDM6B*	739	54.79	539.69
	*TP63*	1199	193.28	515.44
	*THAP1*	607	34.47	509.33
	*EGR1*	786	72.09	507.27

Two-hundred-fifty-base pair sliding window coordinates with association *p* < 0.01 were queried for interval enrichment of ChIP-seq-derived transcription factor binding sites using the ReMap annotation tool. ChIP-seq coordinates were required to have >50% overlap with a sliding window interval. ReMap-derived expected overlaps are obtained from the equivalent number of similarly sized random regions. E-value indicates the expected value, with a higher log-transformed value indicating greater enrichment. Full results are provided in Additional file 1: Table S14

## Discussion

Sleep-disordered breathing is associated with increased risk of a wide range of disorders, including cardiometabolic disease, cancer, cognitive impairment, and interstitial lung diseases, as well as premature mortality [2, 61]. Treatment options, however, are limited by a lack of knowledge of molecular pathways, including those that may be “druggable.” Recent analyses of SDB traits have focused on common variants and identified several preliminary genome-level significant associations [9–11], but did not address gene-based or rare-variant effects. Ten studies and over 21,000 individuals of multiple ancestries with WGS data at unprecedented resolution from the NHLBI TOPMed program combined with densely imputed data from other sources contributed to these results. We identified several variant, gene-based, and pathway-level associations. Analyses adjusted for obesity, a major SDB risk factor, identified loci and genes implicated in pulmonary, inflammatory, and craniofacial pathways. Some associations were population-specific, while others were sex-specific, consistent with population differences and strong sex differences for SDB [20, 62]. Notably, across multiple ancestral groups, we identified a set-based rare-variant association (*p* = 3.48 × 10^−8^) on chromosome X with *ARMCX3*.

### Gene-based results

Across multiple populations, *ARMCX3* (*ALEX3*) and the RNA anti-sense gene *ARMCX3-AS1* were associated with apnea-hypopnea triggered intermittent hypoxia. *ARMCX3* regulates mitochondrial aggregation and trafficking in multiple tissues and facilitates neuronal survival and axon regeneration [63–65]. Wnt signaling regulates reactive oxygen species (ROS) generation and *ARMCX3*-associated mitochondrial aggregation [64, 66]. Potential mechanisms for further study include sensitized carotid body chemoreflexes, interaction with inflammatory mechanisms, and neuronal dysfunction within respiratory centers. Sleep apnea and reduced ventilatory drive are enriched in individuals with a primary mitochondrial disorder [67]. Mitochondria are an important source of ROS, which modulate the acute hypoxic ventilatory response. Mitochondria impact *HIF1A* signaling and may contribute to oxygen sensing [68, 69]. ROS are required for intermittent hypoxia-induced respiratory long-term facilitation [70]. These effects may mitigate the level of hypoxia resulting from recurrent apneas, or conversely, lead to ventilatory instability, promoting apnea occurrence. Mitochondrial ROS also activate the NLRP3 inflammasome in multiple pulmonary diseases, consistent with an inflammation model that includes our IL18-pathway and *HK1* results, ROS-related proinflammatory responses to lung capillary pressure, and evidence of alveolar epithelial injury/SDB interactions [10, 69, 71–73]. Our findings suggest value in investigating the mechanisms by which *ARMCX3* predisposes to SDB, and whether these associations are mediated by neuronal dysfunction and/or ROS and carotid body sensitization, and interact with the inflammasome.

Additional genes were significantly associated in population-specific analyses, including the mitochondrial ribosomal gene *MRPS33.* Mitoribosomes are responsible for the expression of the 13 essential components of the oxidative phosphorylation system, and a majority of the small subunit proteins have been implicated in disease [74]. The expression of several small and large subunit proteins are altered in a hypoxic environment [75]. *MRPS33* expression varies with oxygen treatment in COPD [76].

### Single-variant results

We identified four common frequency associated loci, including multiple-population associations with the *IL18RAP* region. The *IL18RAP* region has been associated with minimum SpO_2_ [10], and here we further identify an association with average event desaturation, highlighting a role in an OSA-specific trait. Multiple variants in this region are also GTEx eQTL variants for both interleukin-18 receptor subunits *IL18RAP* and *IL18R1* (Additional file 1: Table S7) and experimental studies support a role for *IL18* signaling in mediating this association, possibly through effects of pulmonary inflammation on gas exchange (reviewed in [10]).

We identified three population-specific loci, including two novel associations in individuals of European ancestry (Figs. 1 and 2). Sixty-five variants in the *NRG1* region were associated with the AHI (*p* < 1.0 × 10^−8^, Additional file 1: Table S7). This region was suggestively associated with sleep apnea in a Korean population [77]; however, the lead signals appear to be independent (rs10097555 Korean *p* = 2.6 × 10^−6^, EA *p* = 0.91). *NRG1* is associated with lung development and acute lung injury and mediates inflammasome-induced alveolar cell permeability [78–80]. NRG1 promotes accumulation of HIF1A and has increased expression in vascular smooth muscle cells following exposure to intermittent hypoxia [81, 82]. The lead *SLC45A2* region variant rs28777 (average SpO_2_ *p* = 8.08 × 10^−10^) has been associated with multiple traits and is in a splicing regulatory element with extreme population differentiation [83]. An association in the *ATP2B4* region with average SpO_2_ in HAs [9] has been extended to a second hypoxemia trait at the same variant (Per90 *p* = 3.31 × 10^−10^). This gene is the main cellular membrane calcium pump in erythrocytes and also regulates vascular tone [84, 85].

### Pathway analyses

Several gene pathways were identified in EA individuals using imputed gene expression in whole blood (Additional file 1: Table S11). KEGG_RIG_I_LIKE_RECEPTOR_SIGNALING_PATHWAY (retinoic acid-inducible gene I-like) was the most commonly observed, occurring in the top 10 results for 4 of the 5 phenotypes. This pathway initiates the immune response to RNA virus infection [86], consistent with a role for inflammation at the *NRG1* and *IL18RAP* loci. Steroid hormone biosynthesis (the most significantly associated pathway), PPAR signaling, and metabolism (via “starch and sucrose metabolism”) suggest the importance of biological pathways modulating energy homeostasis and balance and metabolic function [87]. In the gene-centric GIGSEA TFBS analysis, V$PEA3_Q6 (*ETV4*) was the lead association for three phenotypes. *ETV4* influences branching in the developing lung and regulates hypoxia-inducible factor signaling [88, 89], a major mechanism influencing ventilatory control.

### Transcription factor binding site enrichment

Several transcription factors were identified through interval enrichment of observed TFBS across the genome (Table 6). *FOXP2* was consistently the most enriched transcription factor and is known to regulate gene expression in epithelial lung tissue and response to lung injury through an inflammatory mechanism [90, 91]. *FOXP2* is also expressed in brainstem respiratory areas including the pre-Bötzinger complex (which is essential for respiratory rhythmogenesis) and impacts airway morphology [92, 93]. Two lysine demethylases (*KDM4B* and *KDM6B*) were also identified. *KDM6B* (*JMJD3*) is required for a functional pre-Bötzinger complex [94, 95] and reduced KDM6B protein expression was reported in hypoxic OSA patients [96]. *Kdm6b* also plays roles in immune function and lung development [97–99]. Drosophila *Kdm4b* knock-outs have increased sleep [100]. *KDM4B* (*JMJD2B*) and *KDM6B* are both members of the JmjC protein domain family and are regulated by *HIF1A*, require oxygen as a cofactor, and act as oxygen sensors for chromatin in hypoxia [101, 102]. *EGR1* mediates hypoxia-induced pulmonary fibrosis [103]. *TP63* is associated with cleft palate in *Tp63* deficient mice, which is associated with an increased prevalence of OSA [104, 105], suggesting that its relationship to OSA may be through pathways influencing craniofacial development. Among the leading 250-base pair sliding window results (Additional file 1: Table S15), 4:105708751-105709001 (Per90 HA *p* = 2.72 × 10^−9^) is of note due to regional associations with lung function and expression in the human lung [106].

### Strengths and weaknesses

This study is the first genome-wide analysis of objectively measured SDB traits using deep sequencing. Together with improved imputation quality, the TOPMed resource has enabled unprecedented genetic resolution. We examined clinically relevant phenotypes measured using rigorous methodology [2, 14]. We analyzed data from 10 studies of individuals from four population groups that used different ascertainment strategies, which may potentially improve the generalization of our results. While this analysis is among the largest performed for SDB traits to date, our moderate sample size has lower power to detect weaker associations, and data were not available to replicate these first rare-variant associations. We did not specifically study the central apnea-hypopnea index due to the relatively low prevalence of central sleep apnea (< 2%) in these largely community-based studies [36, 37]. While there are multiple lines of evidence in the literature to support our findings, additional experimental follow-up analyses are required.

## Conclusions

We have identified the first rare-variant and additional common-variant associations at genome-level significance with objectively measured SDB traits in humans. The results point to biologically relevant pathways for further study, including a novel X-linked association (*ARMCX3*), and a number of associations in genes that modulate lung development, inflammation, respiratory rhythmogenesis, and *HIF1A*-mediated hypoxic-response pathways. These associations will motivate future sample collection and follow-up in cell-line and animal validation studies, with potential therapeutic benefit for sleep-disordered breathing and related comorbidities.

## Supplementary Information

**Additional file 1: Table S1.** NHLBI TOPMed Consortium. **Table S2.** NHLBI TOPMed Consortium Sleep Working Group. **Table S3.** Pairwise Phenotype and Covariate Correlations. **Table S4.** MMSKAT gene-based multiple-population results (*p* < 0.01). **Table S5.** MMSKAT gene-based population-specific results (*p* < 0.01). **Table S6.** Lead MMSKAT result variants. **Table S7.** Single-variant analysis results for lead loci. **Table S8.** Lookups of previously reported GWAS results. **Table S9.** MetaXcan imputed gene expression results. **Table S10.** Lead genes in multiple MetaXcan results. **Table S11.** GIGSEA KEGG pathway results. **Table S12.** GIGSEA MsigDB transcription factor binding site enrichment results. **Table S13.** GIGSEA MsigDB miRNA binding site enrichment results. **Table S14.** Sliding window analysis transcription factor binding analysis enrichment. **Table S15.** Lead sliding window analysis results.

**Additional file 2: Figure S1.** Study Overview. **Figure S2.** NRG1 Locus Models Manhattan and QQ Plots. **Figure S3.** SLC45A2 Locus Models Manhattan and QQ Plots. **Figure S4.** IL18RAP Locus Models Manhattan and QQ Plots. **Figure S5.** ATP2B4 Locus Models Manhattan and QQ Plots.

## Data Availability

Variant-level meta-analysis data are available for visualization and download at the Sleep Disorders Knowledge Portal: https://sleep.hugeamp.org/.

## Abbreviations

AA: African-American
AsA: Asian-American
BMI: Body mass index
CCDG: Centers for Common Disease Genomics
EA: European-American/Australian
GWAS: Genome-wide association study
HA: Hispanic/Latino-American
MAF: Minor allele frequency
OSA: Obstructive sleep apnea
Per90: Percent of the sleep recording with oxyhemoglobin saturation < 90%
ROS: Reactive oxygen species
SDB: Sleep-disordered breathing
SpO_2_: Oxyhemoglobin saturation
TFBS: Transcription factor binding site
TOPMed: Trans-Omics for Precision Medicine
WGS: Whole-genome sequencing
