# Supplementary material for: Whole-genome association analyses of sleep-disordered breathing phenotypes in the NHLBI TOPMed program
Source: Genome Med. 2021 Aug 26;13:136. doi: 10.1186/s13073-021-00917-8 (PMC8394596; doi:10.1186/s13073-021-00917-8)
Supplement: Supplementary file 2 — Additional file 2: Figure S1. Study Overview. Figure S2. NRG1 Locus Models Manhattan and QQ Plots. Figure S3. SLC45A2 Locus Models Manhattan and QQ Plots. Figure S4. IL18RAP Locus Models Manhattan and QQ Plots. Figure S5. ATP2B4 Locus Models Manhattan and QQ Plots. [file 13073_2021_917_MOESM2_ESM.pdf]

Fig. S1: Study Overview

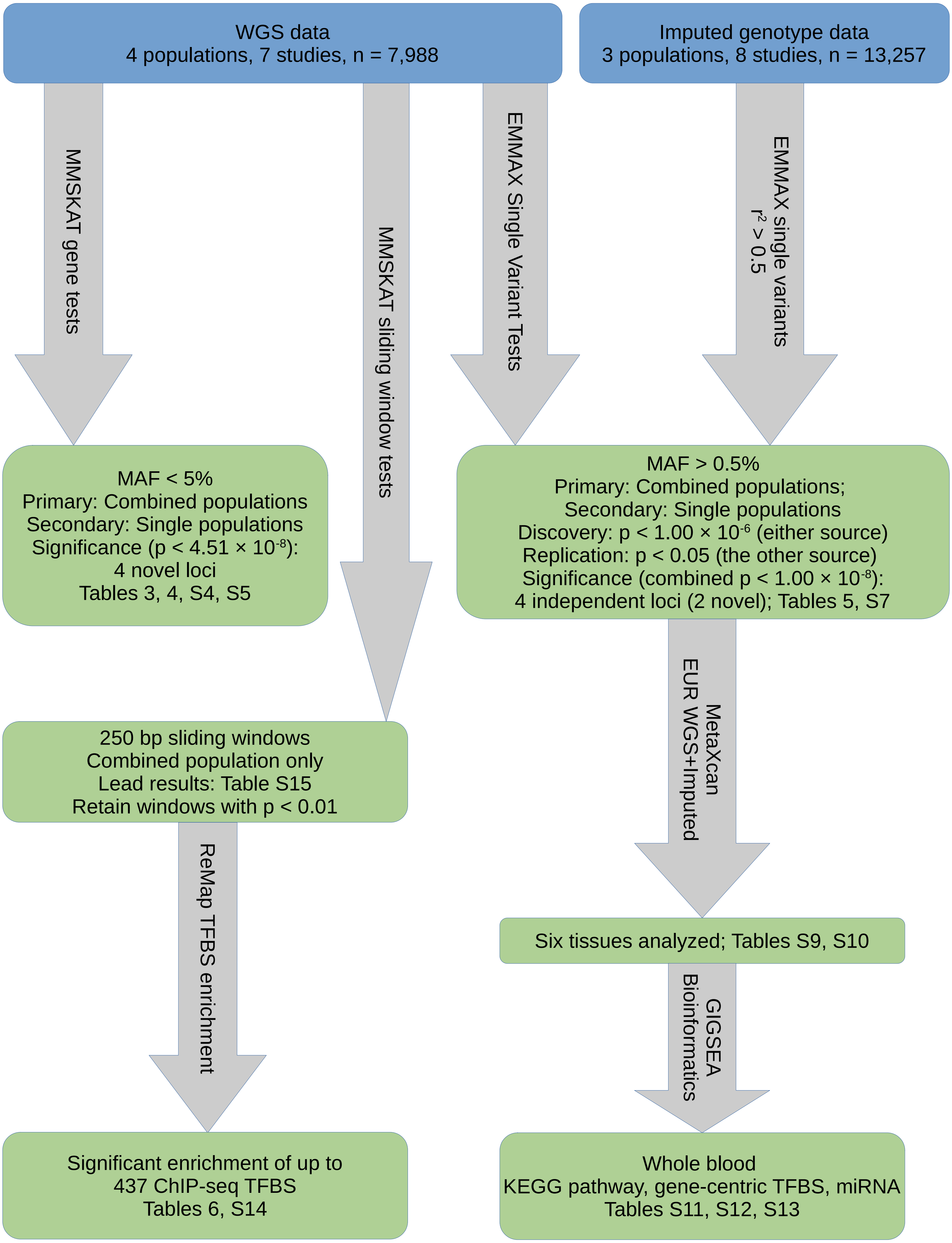

Two main participant data sources were used: whole-genome sequencing (WGS) and imputed genotype data. Gray arrows indicate the main analyses, which are further detailed in the Methods section. Green boxes indicate additional design decisions and which table(s) each analysis is summarized in. There were 4 significant MMSKAT associations (using WGS data), and 4 independent EMMAX GWAS associations (using WGS and imputed genotype data).

Fig. S2: NRG1 Locus Models Manhattan and QQ Plots

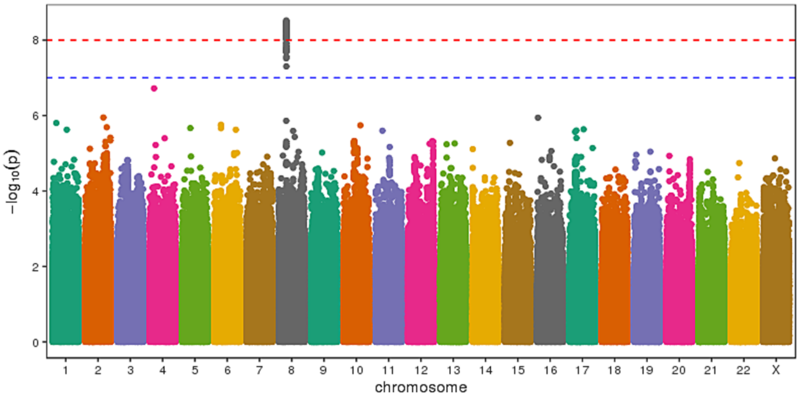

AHI European-American/Australians

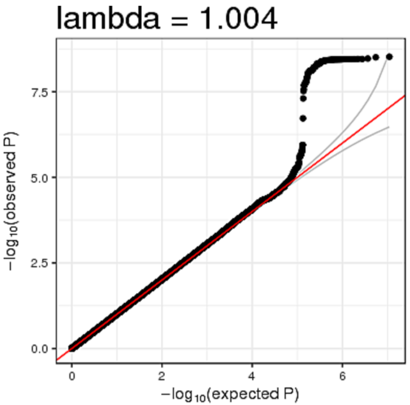

AHI European-American/Australians

AHI analyses of European-Americans and Australians are shown. The Manhattan and QQ plots are based on a meta-analysis of WGS and imputed data. All variants are shown, including variants with low combined p-values that did not meet replication criteria.

Fig. S3: SLC45A2 Locus Models Manhattan and QQ Plots

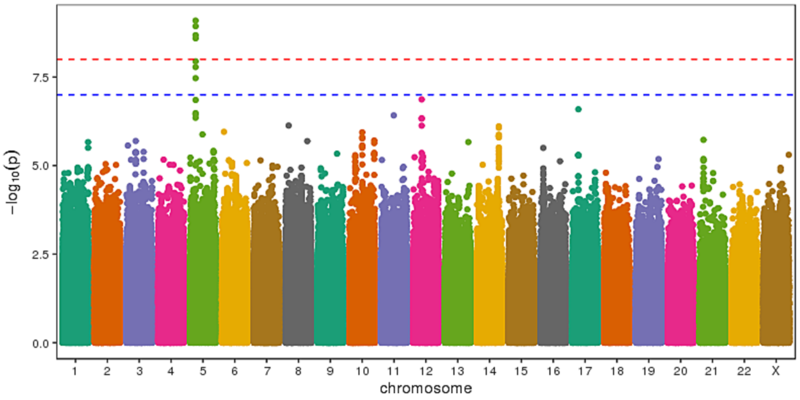

Average SpO2 European-American/Australians

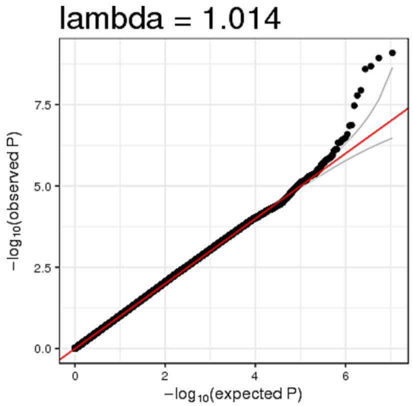

Average SpO2 European-American/Australians

Average SpO2 analyses of European-Americans and Australians are shown. The Manhattan and QQ plots are based on a meta-analysis of WGS and imputed data. All variants are shown, including variants with low combined p-values that did not meet replication criteria.

Fig. S4: IL18RAP Locus Models Manhattan and QQ Plots

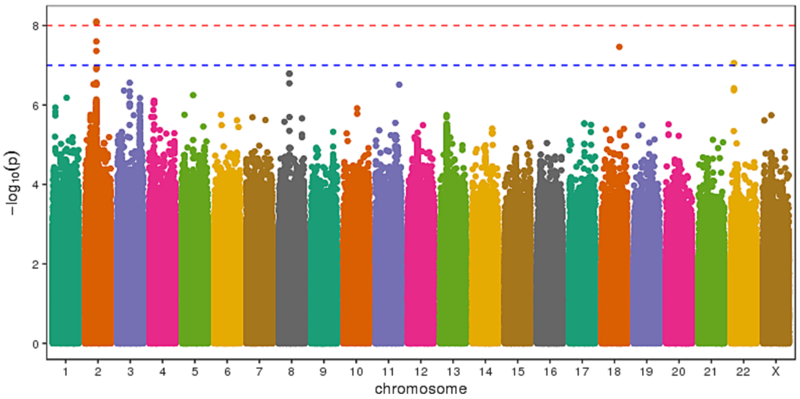

Minimum SpO2 Combined

lambda = 1.011

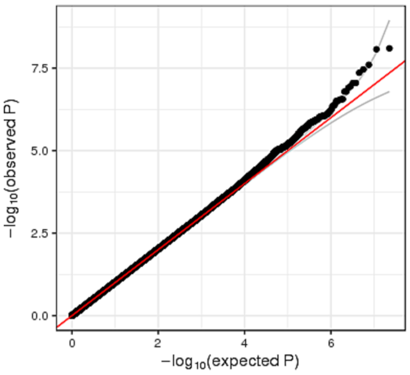

Minimum SpO2 Combined

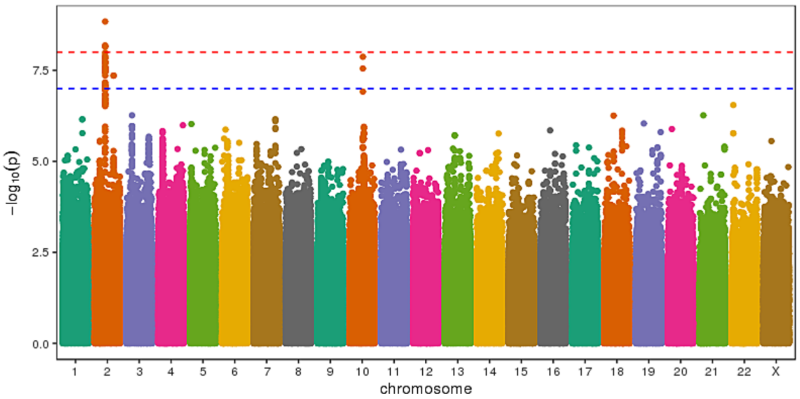

Minimum SpO2 Males

lambda = 1.016

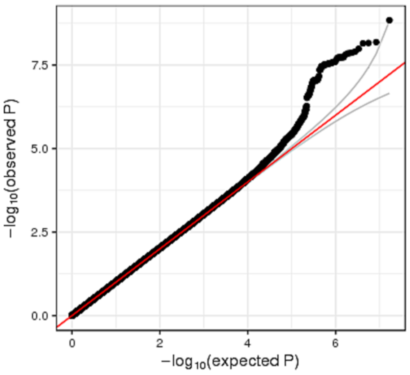

Minimum SpO2 Males

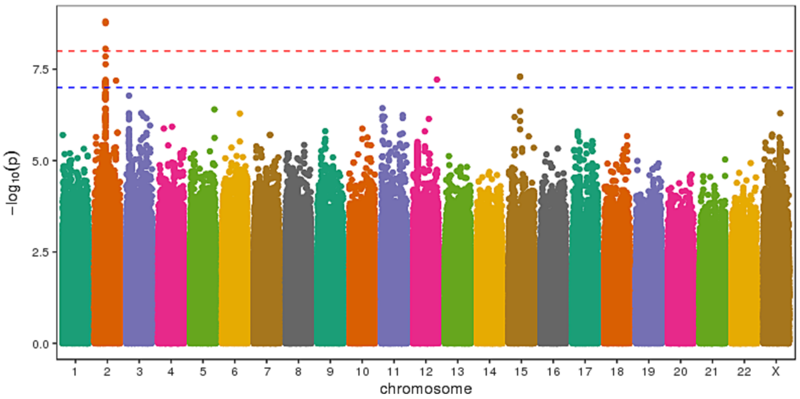

Average Desaturation Combined

lambda = 1.006

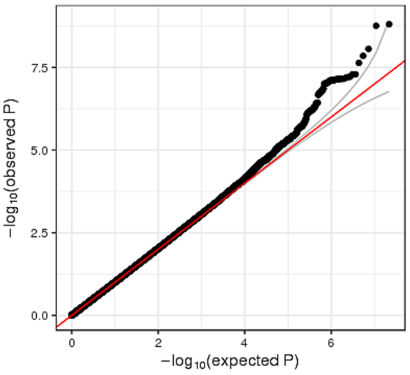

Average Desaturation Combined

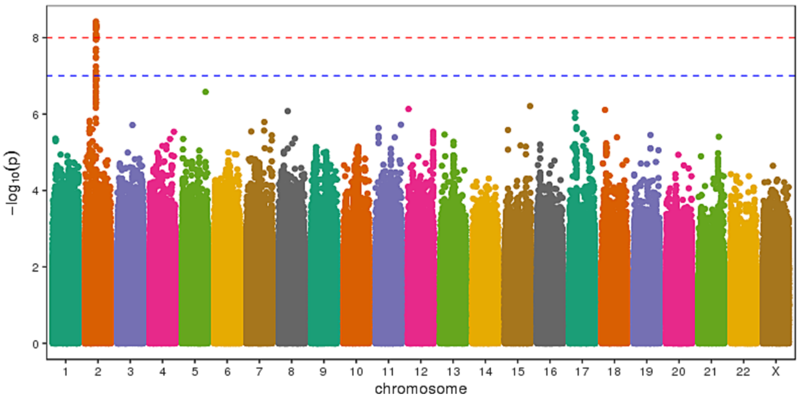

Average Desaturation Males

lambda = 1.004

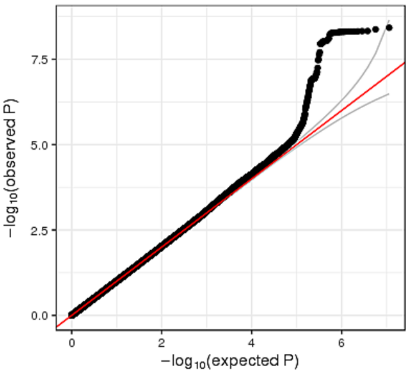

Average Desaturation Males

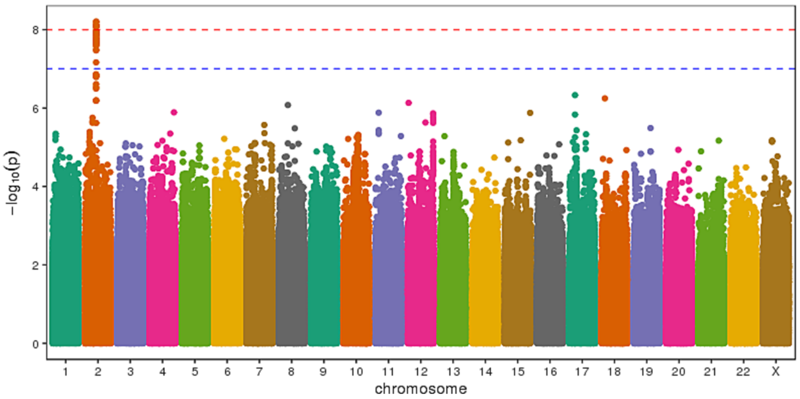

Average Desaturation European-American/Australian Males

lambda = 0.998

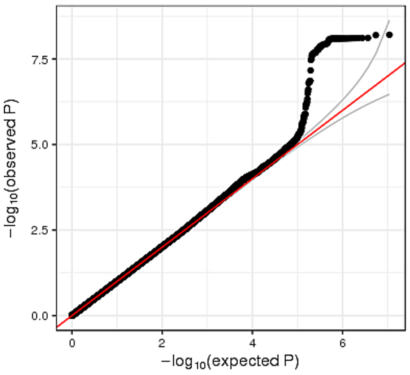

Average Desaturation European-American/Australian Males

All models with significant IL18RAP loci associations are shown. From top to bottom: Minimum SpO2 all individuals), Minimum SpO2 (all males), Average Desaturation (all individuals), and Average Desaturation (all males). Additional European-American Average Desaturation results listed for comparison in Table S6 did not clear the $p < 1e-8$ threshold. The Manhattan and QQ plots are based on a meta-analysis of WGS and imputed data. All variants are shown, including variants with low combined p-values that did not meet replication criteria.

Fig. S5: ATP2B4 Locus Models Manhattan and QQ Plots

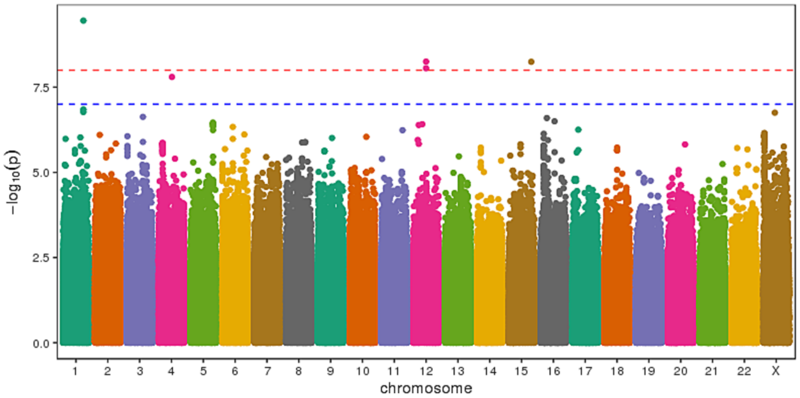

Per90 Hispanic/Latino Americans

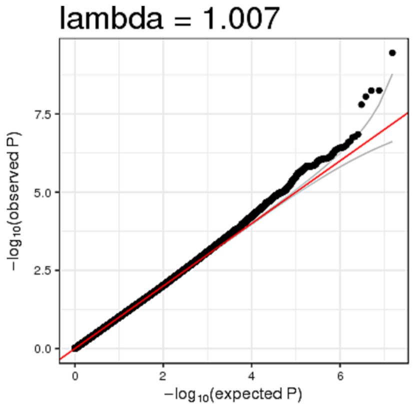

Per90 Hispanic/Latino Americans

Per90 analyses of Hispanic/Latino-Americans are shown. The Manhattan and QQ plots are based on a meta-analysis of WGS and imputed data. All variants are shown, including variants with low combined p-values that did not meet replication criteria.
